# Persistence of Suspected Probiotic Organisms in Preterm Infant Gut Microbiota Weeks After Probiotic Supplementation in the NICU

**DOI:** 10.3389/fmicb.2020.574137

**Published:** 2020-09-25

**Authors:** Efrah I. Yousuf, Marilia Carvalho, Sara E. Dizzell, Stephanie Kim, Elizabeth Gunn, Jennifer Twiss, Lucy Giglia, Connie Stuart, Eileen K. Hutton, Katherine M. Morrison, Jennifer C. Stearns

**Affiliations:** ^1^Department of Pediatrics, McMaster University, Hamilton, ON, Canada; ^2^Centre for Metabolism, Obesity and Diabetes Research, McMaster University, Hamilton, ON, Canada; ^3^Department of Obstetrics & Gynecology, McMaster University, Hamilton, ON, Canada; ^4^Department of Pediatrics, Division of Neonatology, McMaster University, Hamilton, ON, Canada; ^5^Neonatal Follow Up Clinic, McMaster Children’s Hospital, Hamilton, ON, Canada; ^6^Department of Medicine, Farncombe Family Digestive Health Research Institute, McMaster University, Hamilton, ON, Canada

**Keywords:** probiotics, early preterm infants, gut microbiota, *Bifidobacterium*, *Lactobacillus*

## Abstract

Probiotics are becoming a prevalent supplement to prevent necrotizing enterocolitis in infants born preterm. However, little is known about the ability of these live bacterial supplements to colonize the gut or how they affect endogenous bacterial strains and the overall gut community. We capitalized on a natural experiment resulting from a policy change that introduced the use of probiotics to preterm infants in a single Neonatal Intensive Care Unit. We used amplicon sequence variants (ASVs) derived from the v3 region of the 16S rRNA gene to compare the prevalence and abundance of *Bifidobacterium* and *Lactobacillus* in the gut of preterm infants who were and were not exposed to a probiotic supplement in-hospital. Infants were followed to 5 months corrected age. In the probiotic-exposed infants, ASVs belonging to species of *Bifidobacterium* appeared at high relative abundance during probiotic supplementation and persisted for up to 5 months. In regression models that controlled for the confounding effects of age and antibiotic exposure, probiotic-exposed infants had a higher abundance of the suspected probiotic bifidobacteria than unexposed infants. Conversely, the relative abundance of *Lactobacillus* was similar between preterm groups over time. *Lactobacillus* abundance was inversely related to antibiotic exposure. Furthermore, the overall gut microbial community of the probiotic-exposed preterm infants at term corrected age clustered more closely to samples collected from 10-day old full-term infants than to samples from unexposed preterm infants at term age. In conclusion, routine in-hospital administration of probiotics to preterm infants resulted in the potential for colonization of the gut with probiotic organisms post-discharge and effects on the gut microbiome as a whole. Further research is needed to fully discriminate probiotic bacterial strains from endogenous strains and to explore their functional role in the gut microbiome and in infant health.

## Introduction

In healthy full-term infants, bacteria begin to colonize the gut at birth (Perez-Muñoz et al., 2017) and complex microbial communities are formed dynamically as the infant develops (Bäckhed et al., 2015). Preterm birth alters bacterial colonization due to factors including immaturity of the gut environment, exposure to antibiotics, and supplemental feeding with formula (Groer et al., 2014). Infants born preterm are at risk of developing sepsis and necrotizing enterocolitis (NEC), leading causes of mortality and morbidity in this population (Kona and Matlock, 2018). Probiotics, containing strains of *Lactobacillus* sp. and/or *Bifidobacterium* sp. are effective at reducing the incidence of NEC (Deshpande et al., 2010; AlFaleh and Anabrees, 2014; Olsen et al., 2016; Sawh et al., 2016), and may also reduce sepsis in very low birthweight infants (Kona and Matlock, 2018). The mechanisms for how probiotic organisms protect against NEC are largely unknown but may include their ability to increase mucus production, prevent the adherence of enteric pathogens to the gut epithelium (Ewaschuk et al., 2008) and increase barrier function of gut epithelial cells (Mack et al., 1999). Due to their effectiveness against NEC, probiotics are now administered to preterm infants in many neonatal intensive care units (NICUs) around the world. In fact, recent clinical practice guidelines from the American Gastroenterology Association (AGA) suggest that certain probiotic *Lactobacillus* and *Bifidobacterium* strains (and combinations of these strains) be used for the prevention of NEC in preterm and low birth weight infants (Su et al., 2020).

What is not known is whether probiotic strains establish long-term or permanent colonization in the preterm infant gut and if so, what impact such colonization has on infant health and development. Increased DNA from species of *Bifidobacterium* has been seen in preterm infant stool during probiotic supplementation but this often becomes reduced after supplementation has ceased (Li et al., 2004; Mohan et al., 2006; Plummer et al., 2018; Strus et al., 2018). In one small study, a persistent suspected probiotic signal from the genus *Bifidobacterium* (but not *Lactobacillus)* was identified in the post-discharge fecal sample of four infants after discontinuation of a probiotic containing *Bifidobacterium bifidum* and *Lactobacillus acidophilus* (Abdulkadir et al., 2016). No comparator control group was included at the post-discharge timepoint. Therefore, it is unclear if this represented probiotic or endogenous bifidobacterial colonization.

In the current paper, we describe the presence of suspected probiotic bacterial signatures (based on 16S rRNA gene sequences) in the gut of early preterm infants several weeks after discontinuation of the probiotic supplement and investigated the effect of FloraBABY probiotic supplementation on the development of the gut microbiome as a whole. Overall, our results suggest that the administration of probiotic strains to early preterm infants induces earlier colonization by *Bifidobacterium* than would occur in the absence of probiotic supplementation and that this generates a gut microbiome more similar to 10 day old full-term infants.

## Materials and Methods

### Study Participants and Design

Preterm infants were enrolled in the study within 72 h after birth at either McMaster Children’s Hospital or St. Joseph’s Healthcare Hamilton. Exclusion criteria included triplets or higher order multiples and diagnoses of surgical bowel diseases and/or structural bowel abnormalities. Recruitment of 69 preterm infants took place between April 2017 and February 2018. Infants who developed surgical NEC during the study were excluded from further study. Ethics approval for the study was obtained by the Hamilton Integrated Research Ethics Board and parents provided written, informed consent at the time of enrollment.

At enrollment, parents completed a baseline questionnaire on prenatal exposures. Data about the pregnancy and birth and the infant’s in-hospital progress, including nutrition, medication exposure and growth, were collected from antenatal records, birth records, and the maternal and infant charts. Information about infant diet and medication use (including probiotic supplements) following hospital discharge were collected from parents at the first study visit, which took place as close to the term corrected age of 40 weeks as possible, and at 6 weeks, 12 weeks, and 5 months corrected age. These visits took place at McMaster Children’s Hospital (Hamilton, ON, Canada) or at the participant’s home.

In November 2017, there was a practice change in the Neonatal Intensive Care Unit (NICU) of McMaster Children’s Hospital (Hamilton, ON, Canada) resulting in the routine use, of a probiotic treatment for infants born at either less than 34 weeks gestational age or with birthweight less than 2 kg. Infants were excluded from probiotic supplementation if they had any of: congenital gastro-intestinal (GI) anomalies that had not undergone surgical repair, were NPO, had confirmed sepsis, were diagnosed or suspected to have congenital or acquired immunodeficiency syndrome (i.e., HIV, SCID) or had suspected Cow’s Milk Protein Allergy, or other enteropathy. The commercially available FloraBABY probiotic (Renew Life Canada, Brampton ON, Canada) was used. According to the manufacturer, this contains 0.5 g (2 billion CFU bacteria) per single dose sachet, including: *Bifidobacterium breve* (HA-129), *Lactobacillus rhamnosus* (HA-111), *Bifidobacterium bifidum* (HA-132), *Bifidobacterium longum* subsp. *infantis* (HA-116), and *Bifidobacterium longum* subsp. *longum* (HA-135). The FloraBABY supplement was prepared by nursing staff at the infant’s bedside from single dose sachets by mixing with 1 mL of either expressed breastmilk or sterile water. Following introduction, the probiotic was provided daily to the infant until discharge or transfer to another hospital. The study participants for this analysis were a subgroup of infants enrolled in the Baby & Pre-Mi pilot study. Inclusion in this sub-study was based on admission to the NICU at McMaster Children’s Hospital (MUMC) either prior to or after the practice change, gestational age under 32 weeks (early preterm), and collection of stool samples in-hospital and at the term study visit.

### Stool Sample Collection

Following enrollment in the Baby & Pre-Mi study, stool samples were collected every other day until the infant was either discharged from hospital or had reached term corrected age. Diapers with stool were transferred into pre-labeled plastic bags by nursing staff and immediately stored in a −20°C freezer located in the NICU. The sample was then transferred by research personnel to the laboratory and continued to be stored at −20°C until processing. In addition to the stool samples collected in-hospital, samples were also collected at visits that occurred at term, 6 weeks, 12 weeks, and 5 months corrected age. Parents collected the stool sample with supplied, standardized materials and were instructed to store the a sample in a household freezer and then bring the frozen sample to the study visit. If the infant was still in-hospital at the time of the study visit, the sample was collected by the infant’s nurse.

### Term Comparator Cohort

Stool samples collected from a cohort of 51 full-term infants from the Baby & Mi pilot study were utilized for comparison with our early preterm cohort. This study is also a longitudinal, prospective study wherein women with uncomplicated pregnancies were recruited during pregnancy from midwifery practices, and infants born at term (>37 weeks gestation) were subsequently enrolled. Ethics approval for the Baby & Mi study was obtained from all participating sites and parents provided written, informed consent at the time of enrollment. The development of the gut microbiome up to 12 weeks of life for this cohort has been reported elsewhere (Stearns et al., 2017). For inclusion in the comparator group for the current study, infants had to be healthy, vaginally born, been breastfed to at least 5 months and not have received intrapartum antibiotics. 16S rRNA gene data from 199 stool samples collected at 10 days, 6 weeks, 12 weeks, and 5 months of life were used in this analysis.

### DNA Extraction, Sequencing of Bacterial Tags and Analysis

DNA was extracted from 0.1 g of stool with mechanical lysis with 2.8 mm ceramic beads and 0.1 mm glass beads for 3 min at 3000 rpm in 800 μl of 200 mM sodium phosphate monobasic (pH 8) and 100 μl guanidinium thiocyanate EDTA N-lauroylsarcosine buffer (50.8 mM guanidine thiocyanate, 100 mM ethylenediaminetetraacetic acid and 34 mM N-lauroylsarcosine) as previously described (Stearns et al., 2015, 2017). This extract was then purified with the MagMAX-96 DNA Multi-Sample Kit (Life Technologies, Carlsbad, CA) on the MagMAX Express-96 Deep Well Magnetic Particle Processor (Applied Biosystems, Foster City, CA). The DNA was quantified using a NanoDrop 2000c Spectrophotometer (Thermo Scientific, Mississauga, ON Canada). Amplification of the bacterial 16S rRNA gene v3 region (150 base pair) tags was performed as previously described (Bartram et al., 2011) with the following changes: 5 pmol of primer, 200 μM of each dNTP, 1.5 mM MgCl2, 2 μl of 10 mg/ml bovine serum albumin, and 1.25 U Taq polymerase (Life Technologies, Carlsbad, CA, United States) were used in a 50 μl reaction volume. The PCR program used was as follows: 94°C for 2 min followed by 30 cycles of 94°C for 30 s, 50°C for 30 s, and 72°C for 30 s, then a final extension step at 72°C for 10 min. Illumina libraries were sequenced in the McMaster Genomics Facility with 250 base pair sequencing in the forward and reverse directions on the Illumina MiSeq instrument. The completed run was de-multiplexed with Illumina’s Casava software. Adapter, primer and barcode sequences were trimmed from sequencing reads with cutadapt (Martin, 2011) then ASVs were inferred from the sequenced data using the DADA2 pipeline (Callahan et al., 2016). The vegan package (v2.5-6) in R was used to calculate alpha diversity metrics, including observed richness and Shannon diversity index (Oksanen et al., 2018). Observed richness was estimated from ASV counts using the rarefy function, with a sample depth of 5, 000 sequences, while Shannon diversity index was calculated with the diversity function. One preterm infant sample did not meet the sample depth and was not included in observed richness calculations. Beta diversity was based on Bray-Curtis dissimilarity matrices calculated from relative abundance values of all ASVs.

### Bacterial Species Phylogeny

In order to resolve the species distribution of the most abundant ASVs assigned to the *Bifidobacterium* genus, a reference tree was made from reference sequences of *Bifidobacterium* species from the ribosomal database project (RDP) (Cole et al., 2014). All full-length 16S rRNA gene reference sequences for bifidobacterial species (46 in total) from the RDP were aligned using MUSCLE (Edgar, 2004) then used to create an approximate maximum likelihood phylogeny with FastTree (Price et al., 2010) using the Generalized Time Reversible model.

### Statistics

To assess differences between bacterial communities in each sample (beta diversity), principal coordinate analysis plots were generated in the package phyloseq (v30.0). Differences in permutational multivariate analysis of variance (PERMANOVA) of Bray-Curtis dissimilarities with 99, 999 permutation were assessed using the adonis function in the vegan package. Differences in alpha diversity metrics between preterm groups were assessed using linear mixed modeling (lme4 (v1.1-21) and lmerTest (v.3.1-1) packages), with postmenstrual age, cohort, and percent days on antibiotics as fixed effects, and participant as a random effect. Mixed effects models with a negative binomial distribution and log link function were constructed using the package glmmTMB (v0.2.3) to model bacterial abundance data of preterm infants. ASV counts were the response variable, participant was the random effect, and the exposure variables included cohort, postmenstrual age, and percent days on antibiotics. This mixed effect model, including postmenstrual age as the fixed effect and participant as the random effects, was also used to model *Bifidobacterium* abundance in full-term infants. The total number of reads per sample was log-transformed and used as the offset to account for differences in sequencing depth. Comparative analysis was done to look at differences in beta diversity with PERMANOVA using Bray-Curtis distances. Samples were first stratified by collection time point, then pairwise comparisons were completed between preterm groups and the full-term comparator group. Differences with a *p*-value below 0.05 were considered significant.

## Results

### Baseline and Study Visit Characteristics of Probiotic-Exposed and Unexposed Preterm Infants

**FIGURE 1:**
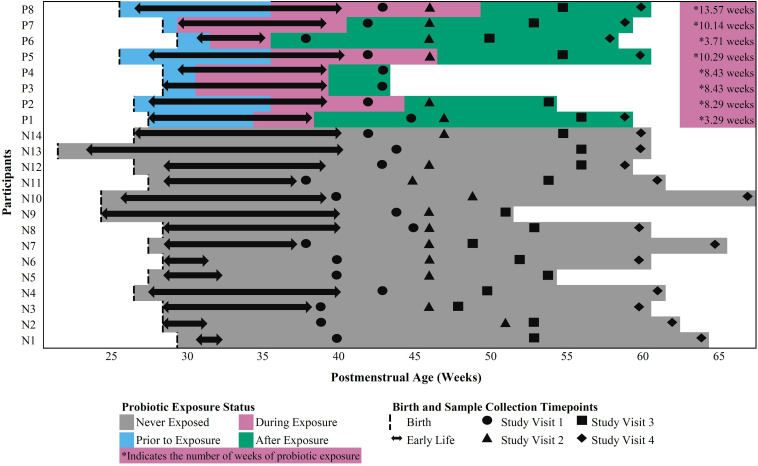
Outline of in-hospital and study visit sample collection across postmenstrual age in probiotic-exposed and unexposed preterm infants.

Twenty-two (22) early preterm infants met the criteria for this study. The gestational age at birth ranged from 22 weeks + 6 days to 30 weeks + 3 days (Table 1). Of these infants, 14 never received the FloraBABY probiotic and 8 infants were enterally administered the probiotic through supplementation of expressed breast milk or sterile water. Infant characteristics were similar between probiotic-exposed and unexposed groups (Table 1). Age of the infant at the time of the first administration of probiotics ranged from 30.29 to 36.14 weeks postmenstrual age. Following this first introduction, infants received the probiotic daily until hospital discharge for a duration of between 3.29 and 13.57 weeks; and postmenstrual age at the time of cessation of probiotic use ranged from 35.86 to 49.57 weeks (Figure 1). A total of 573 stool samples collected during infant hospitalization were included in this analysis. Profiling of 16S rRNA gene was completed in an average (SD) of 26.13 (7.68) samples for each exposed infant and 26.00 (14.58) samples for each unexposed infant. An additional 75 samples were collected at each of the four study visits: term corrected age of 40 weeks (Visit 1), 6 weeks (Visit 2), 12 weeks (Visit 3), and 5 months (Visit 4) corrected age (Figure 1). In the probiotic-exposed cohort, 20 of 25 study visit samples were collected following discontinuation of the probiotic. All infants received breastmilk during hospitalization until at least 37 weeks postmenstrual age (Supplementary Figure S1A) although two infants were weaned from breastmilk during the probiotic supplementation period. Two infants from the probiotic-exposed group did not receive oral or IV antibiotics during their hospitalization and all infants in the unexposed group received antibiotics (Supplementary Figure S1B). All infants that received antibiotics were administered courses of aminoglycoside and *b*-lactam antibiotics during the first 72 h of life. Additionally, 4 infants in the probiotic-exposed group and 12 infants in the unexposed group received additional and variable courses of antibiotics during hospitalization or following discharge (Supplementary Figure S1B).

**TABLE 1:** Participant characteristics of preterm infants.

	Probiotic- exposed (*n* = 8)	Unexposed to probiotic (*n* = 14)	*p*-value
Gestational age at birth, weeks	28.1 ± 1.65	27.5 ± 2.03	0.47
Cesarean delivery, N (%)	6 (75.0%)	10 (71.4%)	0.99
Birth weight, g	975 ± 284	1025 ± 308	0.71
Birth weight z-score	−0.46 ± 0.60	0.12 ± 0.82	0.09
Male, N (%)	3 (37.5%)	4 (28.6%)	0.99
Twins, N (%)	4 (50.0%)	2 (14.3%)	0.14
In-hospital samples collected during FloraBABY supplementation, N (%)	116/209 (55.5%)	0/364 (0%)	**<0.001**
Antibiotic exposure, days (N)	8.63 ± 9.44 (8)	15.3 ± 12.3 (13)	0.21
Weaned from breastmilk, N (%)	6 (75.0%)	7 (50.0%)	0.38
Postmenstrual age (PMA) at weaning from breastmilk, weeks	41.3 ± 3.35	44.8 ± 5.97	0.23

*The data are presented as N (%) for categorical parameters and as mean ± SD for continuous variables. *P*-value < 0.05 using Student’s *t*-test or Mann-Whitney (continuous) or Fisher’s exact test (categorical) were considered to be statistically significant.*

### Comparison of the Relative Abundance of Bifidobacterium ASVs Between Probiotic-Exposed and Unexposed Infants During Hospitalization

We identified a total of 127 ASVs classified to the genus *Bifidobacterium* within our preterm cohort. Of the 457 in-hospital samples collected from all 22 infants in the absence of probiotic exposure (i.e., prior to exposure or in those never exposed), 224 samples from 21 infants had detectable levels of at least one *Bifidobacterium* ASV and this genus made up a mean of 2% of the microbial community. This indicates that *Bifidobacterium* sp. are naturally prevalent, but not abundant in the preterm infant gut microbiome between 1 and 18 weeks postnatally. Four ASVs (ASV 202, ASV 203, ASV 204, and ASV 205) assigned to *Bifidobacterium* sp. had a greater relative abundance than the other bifidobacterial ASVs in our dataset (Supplementary Figure S2). The relative abundance of these four ASVs was 0.005–5% before probiotic exposure and increased to 6–21% after exposure. In contrast, in unexposed infants, the relative abundance of these ASVs was 0.02%. The total relative abundance of all other ASVs belonging to the genus *Bifidobacterium* was below 1.4% in both the probiotic-exposed and unexposed groups, up to the term corrected age visit (Supplementary Figure S2).

### Comparison of the Relative Abundance of Lactobacillus ASVs Between Probiotic-Exposed and Unexposed Infants During Hospitalization

We identified a total of 38 ASVs that were classified to the genus *Lactobacillus* in our preterm cohort. In the absence of probiotic exposure, the average relative abundance of *Lactobacillus* ASVs was 0.9% and the prevalence of these ASVs was 55%. This indicates that *Lactobacillus* sp. were prevalent in the preterm infant gut, yet they were not dominant members of microbial communities profiled in the stool. Of the 38 ASVs belonging to *Lactobacillus*, ASV 2940 had a higher average relative abundance than all other *Lactobacillus* ASVs in probiotic-exposed infants (Supplementary Figure S3) compared to non-exposed preterm infants. During probiotic administration, the prevalence of *Lactobacillus* ASV 2940 increased to 98% with an average relative abundance of 2% of the microbial community (Supplementary Figure S3). From birth to term corrected age (Visit 1), the total relative abundance of all remaining ASVs belonging to the genus *Lactobacillus* remained low.

### Bifidobacterium and Lactobacillus ASV Sequence Identity With Reference 16S rRNA Genes

In order to explore the possibility that the dominant *Bifidobacterium* and *Lactobacillus* ASVs that appeared in probiotic-exposed infants may be the probiotic strains themselves, we determined the sequence identity between ASV sequences and reference sequences of 16S rRNA genes for *Bifidobacterium* and *Lactobacillus* strains from the Ribosomal Database Project (Oksanen et al., 2018). As we did not have the 16S rRNA gene sequence for the commercial FloraBABY product we relied on reference sequences as these can provide an indication of the species classification. We completed a multiple-sequence alignment between the full-length reference 16S rRNA gene sequences and included the short ASV sequences, derived from amplification of the v3 region of the 16S rRNA gene, for all ASVs with an average relative abundance above 1% in the preterm or full-term cohorts that were classified as either *Bifidobacterium* and *Lactobacillus*. We were able to resolve species separation within the *Bifidobacterium* genus with a simple phylogeny, although strain resolution was not possible (Supplementary Figure S4). The four bifidobacterial ASVs that appeared in the probiotic-exposed preterm infants bore the closest sequence similarity to reference sequences of *Bifidobacterium longum* (ASV 202 and 203), *B. bifidum* (ASV 204) and *B. breve* (ASV 205) which matched the species designation of the strains present in the probiotic supplement according to the label. We were unable to resolve species of *Lactobacillus*.

### Impact of Probiotic Supplementation on the Abundance of Bifidobacterium and Lactobacillus After Discontinuation of the Probiotic Supplement

In order to determine the longer-term influence of probiotic supplementation on the abundance of the four bifidobacterial ASVs highlighted above, we examined samples obtained from probiotic-exposed and unexposed preterm infants from term to 5 months corrected age. Negative binomial regression was used to model bacterial count data. Regression models included individual as a random effect and probiotic exposure status, postmenstrual age, and percent of days on antibiotics as fixed effects. To look at the long-term effects of probiotics following discontinuation of use, only samples collected after a minimum 2-week washout period were included in our regression models.

Samples from unexposed infants were found to have a significantly higher percentage of days on antibiotics by the first study visit (*p* = 0.01), and the unexposed infants were on average 3 weeks older at the fourth study visit (*p* = 0.003), compared to the probiotic-exposed infants (Supplementary Table S1). After correcting for antibiotic exposure and repeated sampling, probiotic exposure and postmenstrual age were positively related to the counts of *Bifidobacterium longum* ASV 202, *Bifidobacterium longum* ASV 203, *Bifidobacterium bifidum* ASV 204 and *Bifidobacterium breve* ASV 205 with an interaction effect between age and exposure status (Table 2). Postmenstrual age had a stronger relationship with bifidobacterial abundance in unexposed preterm infants compared to probiotic-exposed infants. Furthermore, the positive relationship of bacterial abundance and postmenstrual age in the unexposed infants suggests that the abundance of bifidobacteria increased naturally over time. In probiotic-exposed preterm infants the *Bifidobacterium* genus and *Bifidobacterium longum* ASV 203 and *Bifidobacterium bifidum* ASV 204 did not increase in abundance over time, while *Bifidobacterium breve* ASV 205 decreased over time. For *Bifidobacterium longum* ASV 202, probiotic exposure and age each had a positive effect (Supplementary Figure S5). Changes in *Bifidobacterium* abundance over time in healthy, vaginally-born full term infants that were breastfed to at least 5 months, and not exposed to the probiotic supplement (*n* = 51) (Stearns et al., 2017) were similarly modeled. The abundance of *Bifidobacterium* in the full-term cohort was not found to be significantly related to postmenstrual age (*p* = 0.06).

**TABLE 2:** Effect of probiotic exposure on the abundance of *Bifidobacterium* and *Lactobacillus* ASVs and genera in preterm infants.

	Cohort†	Postmenstrual	Probiotic-exposed:	Percent days
		age	Postmenstrual age	on antibiotics
	
Taxa	β (95% CI)	β (95% CI)	β (95% CI)	β (95% CI)
*Bifidobacterium*	7.74 (−1.31, 16.8)	0.132 (8.45e-04, 0.263)	−0.122 (−0.0285, 0.0416)	0.00133 (−0.00938, 0.0964)
*Bifidobacteriumlongum* ASV 202	9.84 (0.560, 19.1)*	0.153 (0.0534, 0.252)**	−0.129 (−0.305, 0.0462)	−0.0751 (−0.226, 0.0754)
*Bifidobacteriumlongum* ASV 203	29.0 (10.5, 47.5)**	0.476 (0.295, 0.658)***	−0.487 (−0.845, −0.129)**	−0.00342 (−0.228, 0.159)
*Bifidobacteriumbifidum* ASV 204	15.6 (7.04, 24.2)***	0.233 (0.134, 0.332)***	−0.228 (−0.390, −0.0647)**	−0.0650 (−0.195, 0.0648)
*Bifidobacteriumbreve* ASV 205	21.12 (7.01, 35.4)**	0.243 (0.105, 0.382)***	−0.323 (−0.593, −0.0538)*	−0.160 (−0.367, 0.0480)
Sum of other *Bifidobacterium* ASVs	−0.0484 (−12.8, 12.7)	0.0923 (-0.0268, 0.211)	0.00325 (−0.242, 0.249)	0.0264 (−0.0654, 0.118)
*Lactobacillus*	6.82 (−6.29, 19.9)	0.0785 (−0.0261, 0.183)	−0.117 (−0.371, 0.136)	−0.170 (−0.320, −0.0206)*
*Lactobacillus* ASV 2940	4.50 (−10.2, 19.2)	0.0688 (−0.0494, 0.187)	−0.0723 (−0.355, 0.210)	−0.174 (−0.322, −0.0258)*
Other *Lactobacillus*	−17.0 (−38.9, 4.96)	0.00255 (−0.0689, 0.120)	0.0358 (−0.0738, 0.790)	−0.0243 (−0.457, −0.0282)*

Negative binomial regression was used to model *Bifidobacterium* and *Lactobacillus* count data with an offset for sequencing depth. ^†^Comparison of probiotic-exposed preterm infants to unexposed preterm infants. *p < 0.05. **p < 0.01. ***p < 0.001.

Neither postmenstrual age nor probiotic status were related to the abundance of the *Lactobacillus* ASV 2940, the sum of all other *Lactobacillus* ASVs, or the *Lactobacillus* genus. Antibiotic exposure, however, had a significant negative effect on the abundance of these bacterial groups (Table 2).

### The Effect of Probiotic Persistence on the Gut Microbial Community

The persistence of a suspected probiotic signal in the microbiota of preterm infants begs the question of whether the overall gut microbial diversity and community structure are altered with probiotic administration and how long such an alteration lasts. In order to explore the effects of postmenstrual age, probiotic exposure, and antibiotic exposure on alpha diversity of gut microbial communities in preterm infants following cessation of probiotic use, linear mixed models were used with the individual as a random effect and probiotic exposure, postmenstrual age and percent of days on antibiotics as fixed effects. We found a positive and significant effect of postmenstrual age on species richness, but not Shannon diversity, and no significant relationship was observed between probiotic exposure and alpha diversity metrics (Table 3).

**TABLE 3:** Effect of postmenstrual age, antibiotics, and probiotic exposure on alpha diversity in preterm infants.

	Species richness	Shannon diversity
	β (95% CI)	β (95% CI)
Cohort^†^	30.1 (−14.0, 74.3)	0.00164 (−1.78, 1.83)
Postmenstrual age	0.706 (0.289, 1.11)*	0.00954 (−0.00739, 0.0263)
Probiotic-exposed: Postmenstrual age	−0.505 (−1.35, 0.337)	−0.00169 (−0.0371, 0.0324)
Percent days on antibiotics	−0.254 (−0.870, 0.365)	−0.00759 (−0.0304, 0.0157)

^†^Comparison of probiotic-exposed preterm infants to unexposed preterm infants. *p < 0.05. **p < 0.01. ***p < 0.001.

**FIGURE 2:**
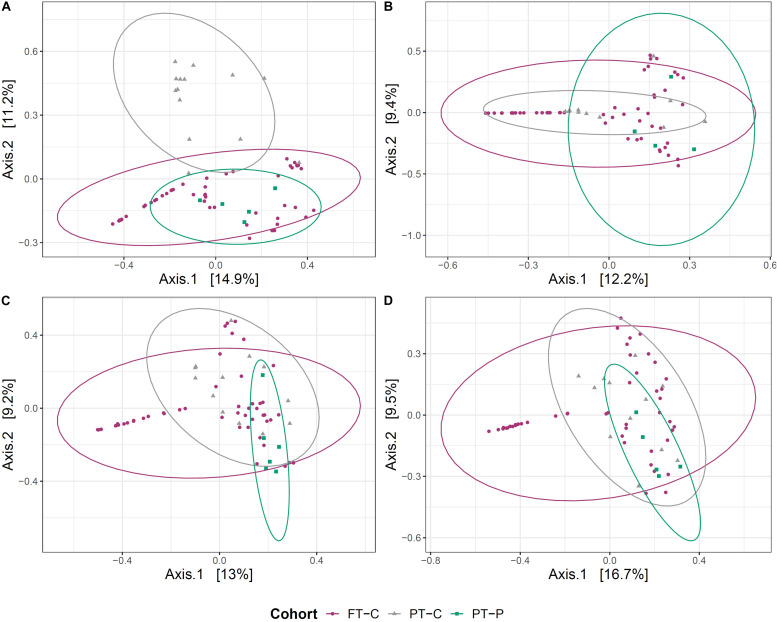
Clustering of the gut microbial community of probiotic exposed preterm infants (after the washout period), unexposed preterm infants and full-term infants. Principal coordinate analysis (PCoA) plots, based on Bray-Curtis dissimilarities of preterm infants not exposed to the probiotic (PT-C), preterm infants exposed to the probiotic (PT-P) and full-term infants (FT-C) at around term **(A)**, 6 weeks **(B)**, 12 weeks **(C)**, and 5 months corrected age **(D)**, compared with samples from full-term infants at 10 days, 6 weeks, 12 weeks, and 5 months postnatal age, respectively.

Beta diversity between probiotic exposed and unexposed preterm infants and full-term infant gut microbiome samples was explored at postmenstrual-matched ages. As with the analyses above, a minimum 2-week washout period from probiotics was used as a cutoff for sample inclusion. First, principal coordinate analysis (PCoA) on Bray-Curtis dissimilarities was used to visualize clustering of samples based on microbial community. After the washout period, the samples collected near term-age from preterm infants exposed to probiotics clustered more closely to samples from 10-day-old full-term infants than did samples at term corrected age from preterm infants never exposed to probiotics (Figure 2A). This clustering did not, however, persist at 6 weeks, 12 weeks or 5 months (Figures 2B–D).

Permutational analysis of variance (PERMANOVA) was used to test the association of variation in microbial communities with cohort, postmenstrual age and, in the case of preterm infants, antibiotic exposure. Antibiotic exposure was not included within models with full-term infants, because only one of 51 infants born full-term had been exposed to antibiotics during the study period. Within preterm infants, at the term corrected age, 20.9% of the variation between gut microbial communities was associated with probiotic exposure (*p* ≤ 0.001) and 11.4% was associated with antibiotic exposure (*p* = 0.006) (Supplementary Table S2). The magnitude of the variance explained by probiotic exposure in preterm infants decreased over time to 13.1% at 6 weeks corrected age, 12.9% at 12 weeks corrected age and 8.5% at 5 months corrected age (Supplementary Table S2). Postmenstrual age only had a significant effect at 5 months of age and was responsible for 11.3% of the variance observed in community structure between preterm groups (*p* = 0.04). When unexposed preterm infants at the term corrected age were compared with 10-day-old full-term infants, 8.7% of the variation in the gut microbiome was associated with cohort, and this proportion decreased over time to 3.9% at 6 weeks, 3.0% at 12 weeks and 2.8% at 5 months (Supplementary Table S2). In contrast, when the probiotic exposed preterm infants at the term corrected age were compared with 10 day old full-term infants, 3.2% of the variation in the gut microbiome was associated with cohort, which changed slightly over time to 2.4% at 6 weeks, 4.0% at 12 weeks and 3.5% at 5 months. This suggests that prior administration of the probiotic had a considerable effect on the gut microbiome of preterm infants, but this effect decreased over the 5-month study period. That said, these findings also highlight that there were significant differences between the gut microbiome of preterm infants at the term corrected age and 10 day-old full-term infants, even when probiotics were administered in early life. At term age, the gut microbiome of probiotic exposed infants was more similar to that of 10-old full-term infants than the gut microbiome of unexposed preterm infants at term age. No significant effect of postmenstrual age or antibiotics was observed on differences in bacterial community structure between preterm and full-term infants.

## Discussion

Infants born very preterm often have a delay in colonization with *Bifidobacterium*, a dominant bacterial genus within the gut microbiome of breastfed full-term infants (Bäckhed et al., 2015; Yassour et al., 2016; Stearns et al., 2017). The delay in the arrival of bifidobacteria may contribute to the establishment of more pathogenic bacteria (Butel et al., 2007) and a susceptibility to sepsis (Mai et al., 2013; Stewart et al., 2017). Routine administration of multi-strain probiotic supplements with *Bifidobacterium* sp. and *Lactobacillus* sp. are effective in reducing the incidence of NEC in preterm infants (Aceti et al., 2015; Chang et al., 2017). However, long-term colonization of the preterm infant gut with bacterial strains from probiotic supplements has not been definitively shown to date. In adults, discontinuing a probiotic reduces the detection of that probiotic signal in stool (Bouhnik et al., 1992; Kullen et al., 1997; Charbonneau et al., 2013), although recently, more variability and possible probiotic colonization of the adult gut has been suggested (Maldonado-Gomez et al., 2016; Zmora et al., 2018). The preterm gut environment could be more permissive to colonization with supplemented bacteria, since the bacteria found there are less abundant and not yet organized into complex communities (Ho et al., 2018). Whether probiotic organisms establish persistent colonization is still unclear, largely due to the fact that molecular profiling of the gut microbiome (e.g., 16S rRNA gene surveys) is unable to distinguish between probiotic and endogenous strains of bacteria.

In this exploratory study, we compared the fecal microbiome of preterm infants exposed and unexposed to probiotics as part of their care following birth. We were able to take advantage of a “natural experiment” that occurred because of a change in clinical practice that stipulated routine probiotic supplementation in this NICU population. The probiotic investigated here (FloraBABY) has been shown to reduce the rate of NEC in a large prospective cohort study (Janvier et al., 2014), although the effect of this probiotic on gut microbial composition has not been previously explored. We also compared preterm cohorts with a cohort of full-term infants that followed the same longitudinal data collection protocol out to 5 months of age. Samples were collected according to postnatal age in full-term infants and corrected age in preterm infants to reflect current pediatric guidelines that recommend preterm infant growth be modeled after healthy term-born infants (American Academy of Pediatrics, 1977). We set out to determine whether probiotic strains given as a supplement were colonizing the preterm infant gut, and to determine if probiotic supplementation exerted a consistent effect on the overall gut microbiome in the post-discharge period. Our results suggest that the administration of FloraBABY to preterm infants increases the abundance of *Bifidobacterium* but not *Lactobacillus* in the infant gut for many weeks after the discontinuation of the probiotic. Further, the gut microbiome at term corrected age in probiotic-exposed preterm infants more closely resembled that of 10-day-old full-term infants than unexposed preterm infants.

In the absence of probiotic exposure, *Bifidobacterium* sp. abundance in our preterm cohort was low during the first months of life, consistent with previous studies (Stewart et al., 2015; Patel et al., 2016; Butcher et al., 2018). Probiotic exposure was associated with a higher abundance of *Bifidobacterium* and *Lactobacillus* in the stool. Increased relative abundance of four ASVs belonging to the genus *Bifidobacterium* (ASV 202 - ASV 205) and one ASV assigned to the genus *Lactobacillus* (ASV 2940) coincided with the period of probiotic administration (Supplementary Figures S2, S3). The suspected probiotic bifidobacterial strain ASVs bore sequence similarity to reference sequences similar to species listed in the ingredients of the probiotic supplement (e.g., *B. longum*, *B. breve*, and *B. bifidum*; Supplementary Figure S4) and were distinct from other naturally occurring strains of *Bifidobacterium* that were present during hospitalization in the absence of probiotic supplementation. Although we were able to discriminate between suspected endogenous bifidobacterial ASVs and suspected probiotic bifidobacterial ASVs at early timepoints in the preterm infant samples, we found that the amplicon-based profiles, from short sequences of the 16S rRNA gene, were unable to discriminate between probiotic-derived and some suspected endogenous strains of *B. longum*, *B. breve* and *B. bifidum* that appeared naturally in unexposed preterm infants beginning at 6 weeks corrected age. Species-level resolution could not be obtained for the suspected probiotic *Lactobacillus* strain ASV. Our data also indicated the appearance of suspected probiotic *Bifidobacterium* ASVs in some infants prior to the start of probiotic supplementation (Supplementary Figures S2A,C,D), suggesting potential cross-colonization within the NICU. This type of cross-colonization has been suspected before in large randomized controlled trials (Costeloe et al., 2016; Plummer et al., 2018).

Both probiotic exposure and postmenstrual age were directly related to increased abundance of *Bifidobacterium* ASVs in preterm infants (Table 2). Previous studies have shown that colonization of the preterm infant gut by *Bifidobacterium* is dependent on postmenstrual age (Butel et al., 2007; Korpela et al., 2018) and that daily probiotic administration until 34 weeks postmenstrual age can increase the *Bifidobacterium* abundance in preterm infants compared to control groups (Watkins et al., 2019). Here we confirm the effect of postmenstrual age on the abundance of bifidobacteria and demonstrate that probiotic exposure increased the initial abundance of bifidobacteria in preterm infants. In the case of *Bifidobacterium longum* ASV 202 this resulted in higher abundance beyond the supplementation period (Supplementary Figure S5), suggesting long-term colonization with this probiotic strain. In contrast, neither postmenstrual age nor probiotic-exposure status were associated with the abundance of *Lactobacillus*, similar to previous studies in preterm infants and adults (Costa et al., 2014; Abdulkadir et al., 2016). It should be noted, however, that stool analysis may underestimate probiotic *Lactobacillus* colonization, as *Lactobacillus* colonizes the small intestine (Hao and Lee, 2004) and attaches to colonic mucosae *in vivo* (Alander et al., 1999). While antibiotic exposure was not found to have a significant effect on the abundance of bifidobacteria; a negative and significant effect of exposure to antibiotics was observed for *Lactobacillus* in our study, which was similar to murine models of early-life antibiotic administration (Cox et al., 2014).

Probiotic exposure was shown to impact the microbial community structure within the preterm infant gut. No differences were observed in alpha diversity measures between probiotic-exposed and unexposed infants, however, beta diversity analysis indicated that samples from probiotic-exposed preterm infants at term age were found to cluster more closely with 10-day old full-term infants than did samples from unexposed preterm infants (Figure 2A). The clustering was not observed at later time points.

Our findings suggest that probiotic supplementation in preterm infants may promote an earlier convergence to an intestinal microbiome that is more similar to healthy, full-term infants; however, more research is needed to determine if probiotic strains of bacteria offer all of the same benefits as endogenous bacteria. More research is also needed to examine the dosage and the length of administration needed to achieve, or avoid, colonization in preterm infants. Furthermore, the influence of antibiotic exposure and breastfeeding on colonization needs to be studied. In our study, all infants were receiving breastmilk at the time of the introduction of the probiotic, with two infants being weaned from breastmilk while continuing to receive the probiotic (Supplementary Figure S1). Breastfeeding is important to the establishment of the microbiota in the infant gut (Bäckhed et al., 2015) and may be an important modifier to the establishment of probiotic strains in the gut. While we have demonstrated that probiotic administration had an effect on the preterm infant gut microbiome, it is still unclear whether probiotic strains colonized the preterm gut long-term. Better strain resolution, through longer read technology or cultured isolates, is needed to track the persistence of probiotic strains in the gut as preterm infants age. If probiotic bacteria can colonize the preterm infant gut, then questions remain about which strains are the most beneficial to infants during this critical stage in development (Ewaschuk et al., 2008).

Strengths of our study include the prospective design, high-resolution longitudinal collection of samples at frequent timepoints, and the quality of clinical data collected from our study population. The policy change within NICU to administer probiotics to all preterm infants born at less than 34 weeks gestation, instead of based on the clinical condition of the infants, created a natural experiment that was also a strength. Limitations of our study included: the small number of preterm infants and the variable timing of sample collection, a limited ability to explore the influence of antibiotic type and dosages on the microbiome., the lack of a placebo-control to account for the prebiotic effect of maltodextrin (Yeo and Liong, 2010) in the FloraBaby supplement, and the limited resolution of ASVs to discriminate probiotic strains of *B. longum*, *B. bifidum*, and *B. breve* from endogenous ones.

Early preterm infants are known to have delays in *Bifidobacterium* colonization compared to infants born full-term. Our results show that enteral administration of a multi-strain probiotic to early preterm infants during hospitalization results in the increased abundance of suspected probiotic bifidobacterial ASVs up to 5 months post-supplementation, and potential induction of probiotic colonization of the infant gut. This increase in *Bifidobacterium* may be related to the potential role of probiotics in reducing NEC development in preterm infants. Further research is needed to identify these probiotic strains and explore their functional role in microbiome development and infant health.

## Membership of the Baby & MI Study Group Members

Alison C. Holloway, Department of Obstetrics and Gynecology, McMaster University, Hamilton, ON, Canada. Helen McDonald, McMaster Midwifery Research Centre, McMaster University, Hamilton, ON, Canada. Elyanne M. Ratcliffe, Department of Pediatrics, McMaster University, ON, Canada. Jonathan D. Schertzer, Farncombe Family Digestive Health Research Institute, McMaster University, Hamilton, Canada. Mike G. Surette, Department of Biochemistry and Biomedical Sciences, McMaster University, Hamilton, Canada. Lehana Thabane, Department of Clinical Epidemiology and Biostatistics, McMaster University, Hamilton, Canada. Andrea Mousseau, Department of Obstetrics and Gynecology, Grand River Hospital Corporation, Kitchener, Canada.

## Data Availability Statement

The datasets presented in this article are not readily available due to the potentially identifiable nature of the data and privacy concerns by study participants. Requests to access the datasets should be directed to JS, stearns@mcmaster.ca.

## Ethics Statement

The studies involving human participants were reviewed and approved by Hamilton Integrated Research Ethics Board, Hamilton Health Sciences Faculty of Health Sciences Research Ethics Board, St. Joseph’s Healthcare Hamilton Research Ethics Board, and Joseph Brant Hospital Research Ethics Board. Written informed consent to participate in this study was provided by the participants’ legal guardian/next of kin.

## Author Contributions

JS, EY, KM, and EH conceived the research objectives. JS, EY, KM, EH, JT, LG, and CS designed the methodology. MC, SD, SK, and EG recruited participants and collected samples and clinical information. EY and JS analyzed the data and wrote the manuscript. All authors edited the manuscript and approved the final draft.

## Conflict of Interest

The authors declare that the research was conducted in the absence of any commercial or financial relationships that could be construed as a potential conflict of interest.

## Abbreviations

NICU: neonatal intensive care unit
NEC: necrotizing enterocolitis
ASV: amplicon sequence variant.
