# Supplementary material for: Persistence of Suspected Probiotic Organisms in Preterm Infant Gut Microbiota Weeks After Probiotic Supplementation in the NICU
Source: Front Microbiol. 2020 Sep 25;11:574137. doi: 10.3389/fmicb.2020.574137 (PMC7552907; doi:10.3389/fmicb.2020.574137)
Supplement: Supplementary file 1 [file Data_Sheet_1.PDF]

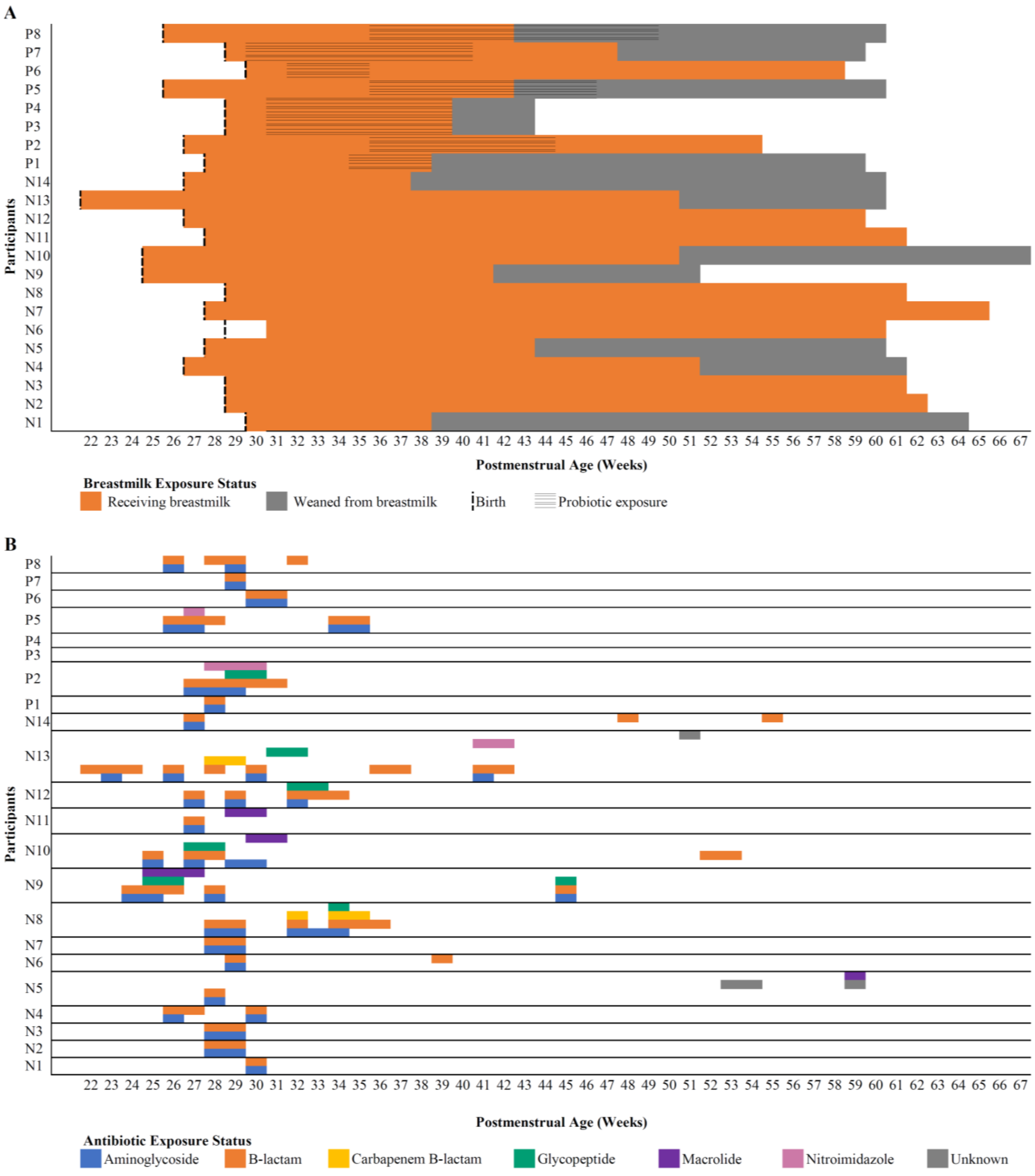

Figure S1. Breastfeeding and antibiotic exposure in preterm infants. Outline of breastfeeding status of probiotic-exposed and unexposed preterm infants depicted over post-menstrual age **(A)** and antibiotic exposure depicted according to antibiotic type over postmenstrual age **(B)**.

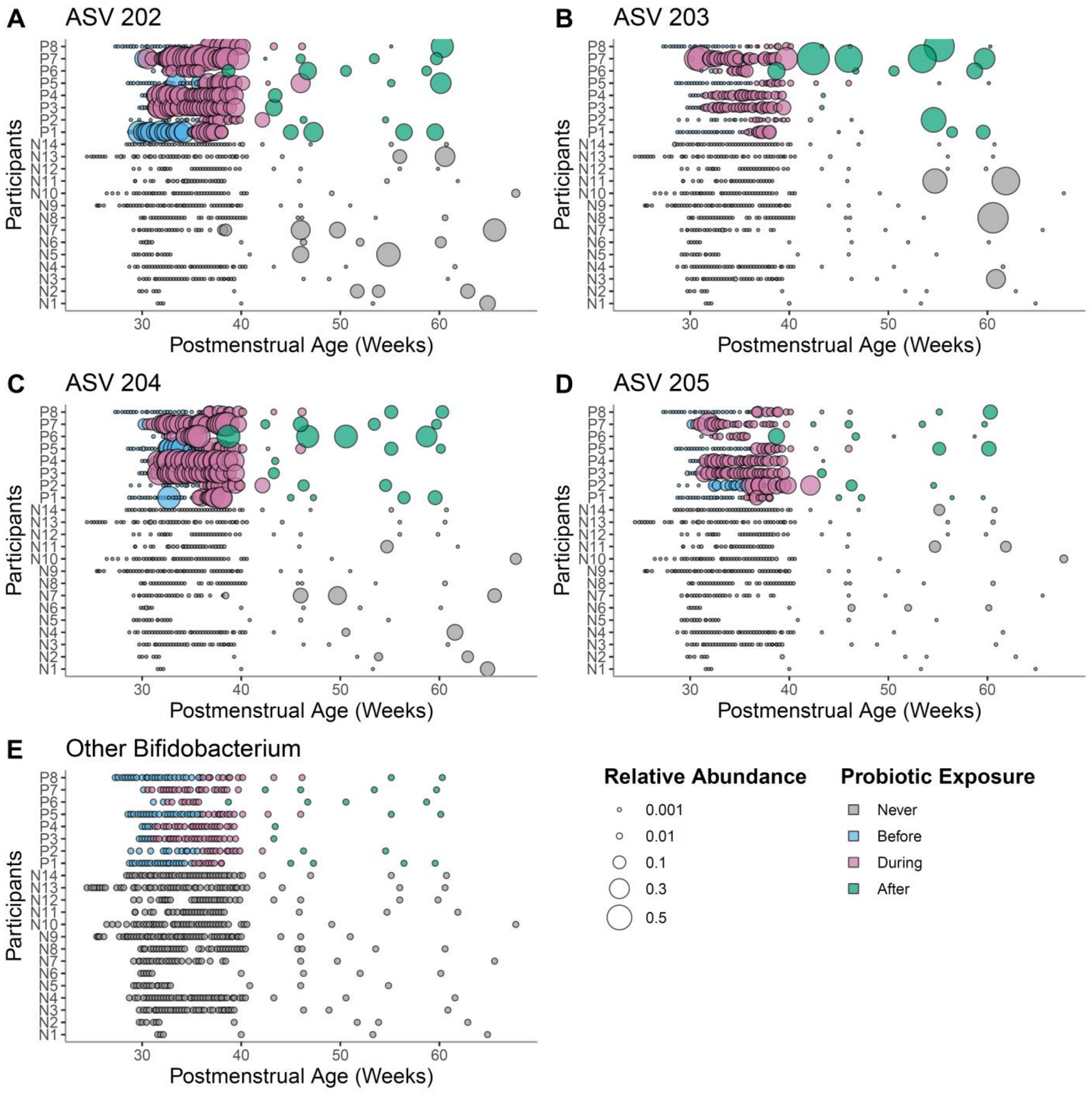

Figure S2. Relative abundance over time of ASVs from the genus *Bifidobacterium* in preterm infants, including suspected probiotic *Bifidobacterium* ASV 202 **(A)**, ASV 203 **(B)**, ASV 204 **(C)**, and ASV 205 **(D)**, as well as the sum of all other *Bifidobacterium* ASVs suspected to be non-probiotic strains **(E)**. Relative abundance is depicted over postmenstrual age at the time of sample collection. Samples were collected from enrollment to the 5 month corrected age study visit for probiotic-exposed (n=8) and unexposed (n=14) preterm infants. Samples are colour-coded according to exposure status: grey (never exposed), blue (prior to exposure), red (during exposure), green (after exposure).

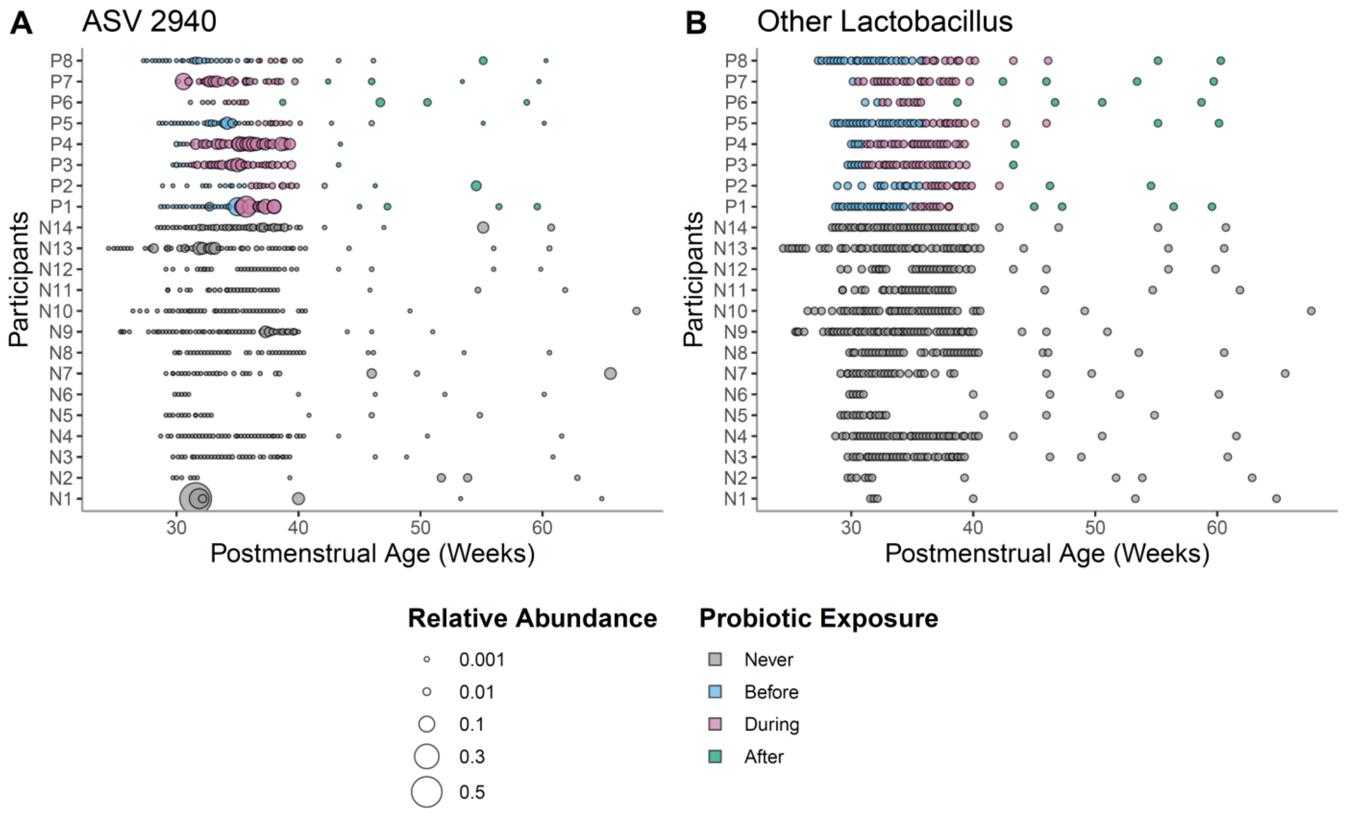

Figure S3. Relative abundance over time of ASVs from the genus Lactobacillus in preterm infants, including suspected probiotic Lactobacillus ASV 2940 **(A)**, and the sum of all other Lactobacillus ASVs suspected to be non-probiotic strains **(B)**. Samples are colour-coded according to exposure status: blue (prior to exposure), red (during exposure), and green (after exposure).

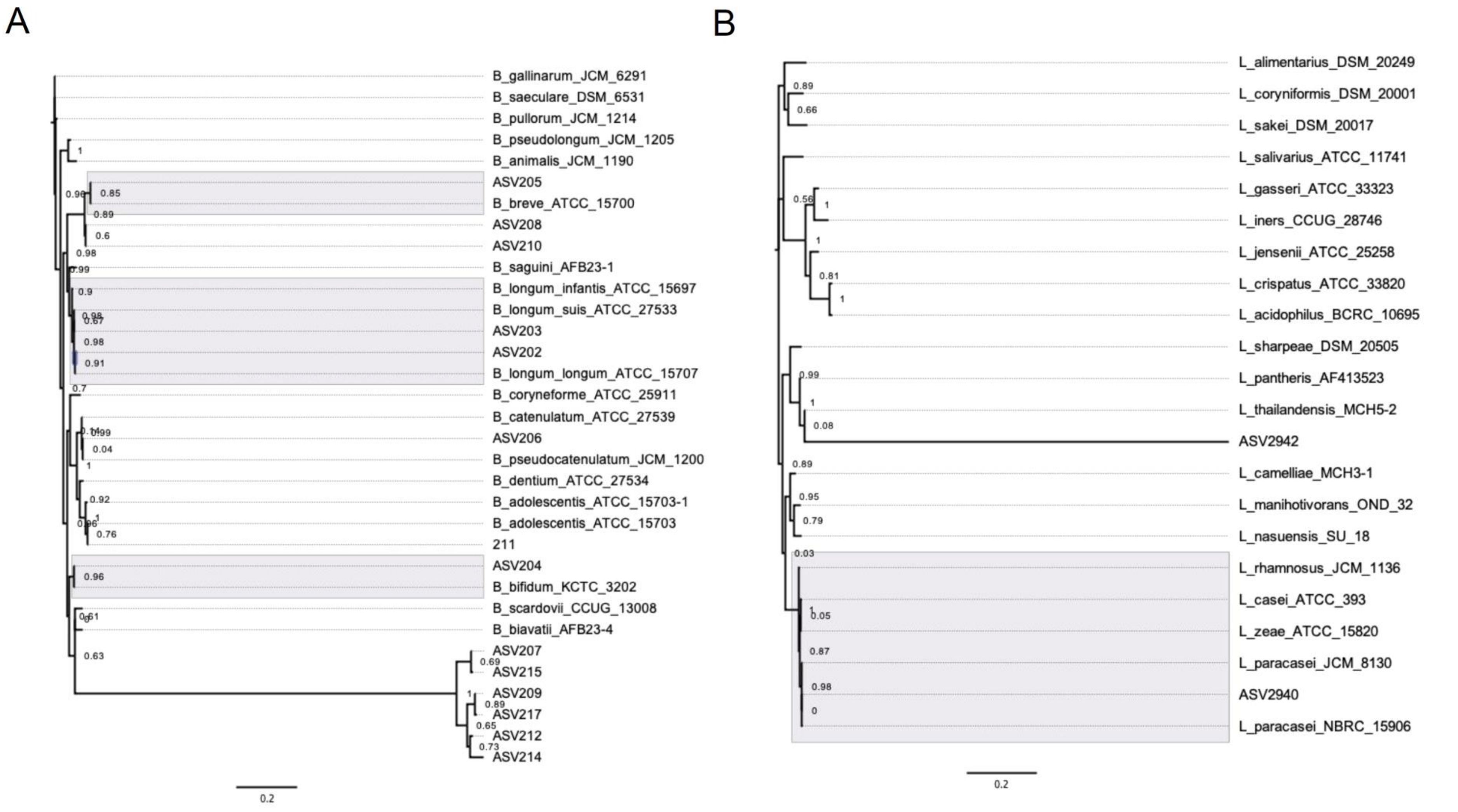

Figure S4. *Bifidobacterium* and *Lactobacillus* diversity in the gut microbiome of preterm infants. Approximate maximum-likelihood trees based on 16S rRNA gene reference sequences and ASV sequences above 1% relative abundance in this study. Reference sequences for members of the genera *Bifidobacterium* **(A)** and *Lactobacillus* **(B)**. The ASVs suspected to be of probiotic origin are highlighted in grey.

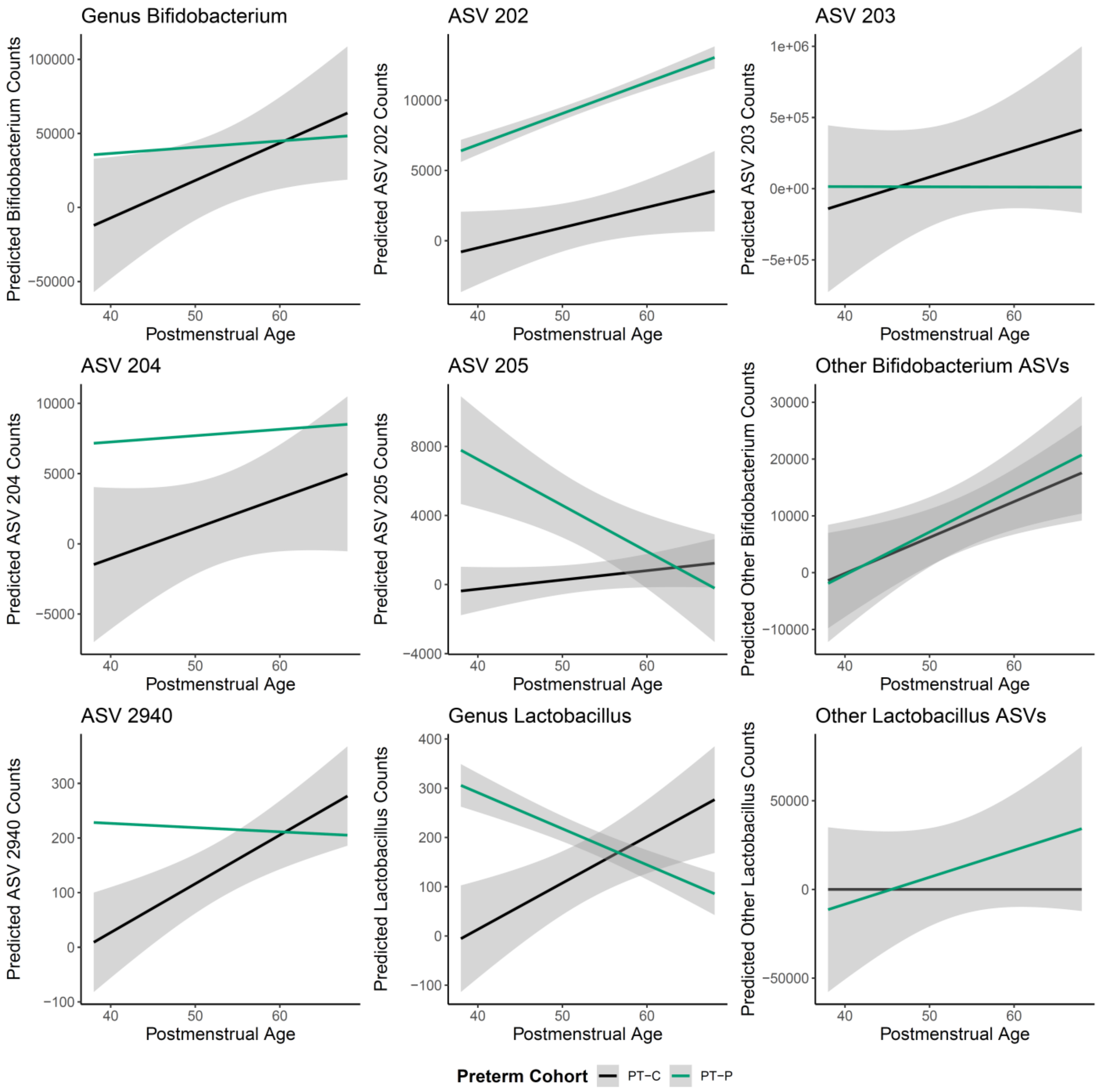

Figure S5. Interaction effect of cohort and postmenstrual age on the abundance of Bifidobacterium and Lactobacillus in preterm infants. Predicted bacterial counts from regression models are compared between unexposed preterm infants (PT-C) and probiotic-exposed preterm infants (PT-P). The grey shaded area represents the 95% confidence interval.

Table S1: Weaning status and percent days on antibiotics for probiotic-exposed infants following cessation of probiotic use and unexposed preterm infants.

		Probiotic-exposed infants following cessation of probiotic use	Unexposed to probiotic	P value
Percentage of days on antibiotics at sample collection, (N)	Study Visit 1	3.0 ± 4.2 (5)*	11.6 ± 8.0 (14)	0.0098
	Study Visit 2	6.9 ± 7.8 (4)	9.2 ± 7.0 (10)	0.6028
	Study Visit 3	5.4 ± 4.5 (6)	6.8 ± 5.6 (12)	0.6165
	Study Visit 4	3.2 ± 2.4 (5)	5.8 ± 4.8 (11)	0.2878
Weaned at sample collection, N (%)†	Study Visit 1	3/5 (60.0%)	3/14 (21.4%)	0.2621
	Study Visit 2	1/4 (25.0%)	3/11 (27.3%)	>0.9999
	Study Visit 3	4/6 (66.7%)	5/13 (38.5%)	0.3498
	Study Visit 4	4/5 (80.0%)	5/12 (41.7%)	0.2941
Postmenstrual age (PMA) at sample collection, weeks (N)	Study Visit 1	42.57 ± 2.10 (5)	41.38 ± 2.28 (14)	0.3459
	Study Visit 2	46.57 ± 0.49 (4)	46.95 ± 1.75 (11)	0.5590
	Study Visit 3	54.03 ± 1.98 (6)	53.04 ± 2.30 (13)	0.3165
	Study Visit 4	59.53 ± 0.52 (5)*	62.26 ± 2.39 (12)	0.0026

The data are presented as N (%) for categorical parameters and as mean ± SD (N) for continuous variables. Continuous variables collected at study visits include (N) to indicate the number of individuals with information collected at each time point. Significant differences between cohorts are indicated by * ($p<0.05$, Student's t-test or Mann-Whitney (continuous) or Fisher's exact test (categorical)).

Table S2: Permutational multivariate analysis of variance (PERMANOVA) exploring the differences in community composition (as assessed using Bray-Curtis dissimilarity matrices) between preterm and full-term infant cohorts.

	Cohort		Postmenstrual age		Percent days on antibiotics	
Subgroup	Variance explained (R^2)	Pr(>F)	Variance explained (R^2)	Pr(>F)	Variance explained (R^2)	Pr(>F)
PT-C vs PT-P						
Term	0.209	**<0.001**	0.0328	0.71	0.114	**0.006**
6 weeks	0.131	0.07	0.0569	0.71	0.0864	0.25
12 weeks	0.129	**0.007**	0.0561	0.41	0.0487	0.57
5 months	0.0847	0.17	0.113	**0.04**	0.0538	0.60
PT-P vs FT-C						
Term	0.0325	**0.02**	0.0117	0.88	NA	NA
6 weeks	0.0238	0.09	0.0181	0.42	NA	NA
12 weeks	0.0400	**0.002**	0.0211	0.22	NA	NA
5 months	0.0349	**0.02**	0.0241	0.17	NA	NA
PT-C vs FT-C						
Term	0.0871	**<0.001**	0.00827	0.95	NA	NA
6 weeks	0.0386	**<0.001**	0.0142	0.59	NA	NA
12 weeks	0.0307	**0.006**	0.0109	0.91	NA	NA
5 months	0.0275	**0.04**	0.0215	0.16	NA	NA
